# How does social accountability contribute to better maternal health outcomes? A qualitative study on perceived changes with government and civil society actors in Gujarat, India

**DOI:** 10.1186/s12913-018-3453-7

**Published:** 2018-08-22

**Authors:** Mukesh Hamal, Tjard de Cock Buning, Vincent De Brouwere, Azucena Bardají, Marjolein Dieleman

**Affiliations:** 1 Athena Institute for Research on Innovation and Communication in Health and Life Sciences (VU University), De Boelelaan 1085, 1081 HV Amsterdam, The Netherlands; 2 ISGlobal, Barcelona Centre for International Health Research (CRESIB), Hospital Clínic-Universitat de Barcelona, Barcelona, Spain; 3 Maternal and Reproductive Health, Department of Public Health, Institute of Tropical Medicine, Antwerp, Belgium; 4 KIT Health, PO Box 95001, 1090 HA Amsterdam, The Netherlands

**Keywords:** Social accountability, Health system, Maternal health, Gujarat, India, Qualitative

## Abstract

**Background:**

Social accountability mechanisms have been highlighted as making a contribution to improving maternal health outcomes and reducing inequities. But there is a lack of evidence on *how* they contribute to such improvements. This study aims to explore social accountability mechanisms in selected districts of the Indian state of Gujarat in relation to maternal health, the factors they address and how the results of these mechanisms are perceived.

**Methods:**

We conducted qualitative research through in-depth interviews and focus group discussions with actors of civil society and government health system. Data were analyzed using a framework of social determinants of maternal health in terms of structural and intermediary determinants.

**Results:**

There are social accountability mechanisms in the government and civil society in terms of structure and activities. But those that were perceived to influence maternal health were mainly from civil society, particularly women’s groups, community monitoring and a maternal death review. The social accountability mechanisms influenced structural determinants – governance, policy, health beliefs, women’s status, and intermediary determinants – social capital, maternal healthcare behavior, and availability, accessibility and the quality of the health service delivery system. These further positively influenced the increased use of maternal health services. The social accountability mechanisms, through the process of information, dialogue and negotiation, particularly empowered women to make collective demands of the health system and brought about changed perceptions of women among actors in the system. It ultimately improved relations between women and the health system in terms of trust and collaboration, and generated appropriate responses from the health system to meeting women’s groups’ demands.

**Conclusion:**

Social accountability mechanisms in Gujarat were perceived to improve interaction between communities and the health system and contribute to improvements in access to and use of maternal health services. The influence of social accountability appeared to be limited to the local/district level and there was lack of capacity and ownership of the government structures.

## Background

There are inequities in maternal health worldwide, both across countries and among sub-populations within them, including India [1–3]. These inequities are revealed in terms of the maternal mortality ratio (MMR) and the uptake of obstetric care or maternal health services, particularly antenatal care (ANC), giving birth in a medical institution and postnatal care (PNC). For example, the MMR (per 100,000 live births) is higher in north Indian states, such as Assam (328) or Uttar Pradesh/Uttarakhand (292) than in southern states, such as Kerala (60) or Tamil Nadu (90) [4], while for other states it falls in between (e.g. Gujarat (122)). The MMR is also higher among the poor, illiterate and scheduled tribes/scheduled castes (SC/ST), and the uptake of maternal health services is lower among these groups [5].

Studies in India have highlighted that lack of accountability in the health system can lead to maternal inequities and deaths [6–8]. Accountability is basically the obligation of an individual or agency to provide information, explain and justify their conduct to stakeholders, backed up with the imposition of sanctions for non-compliance and/or inappropriate behavior [9]. Accountability problems in the health system – such as distorted accountability mechanisms, or unaccountable behavior of health providers and managers – result in its failure to guarantee the availability and functioning of obstetric care services at different levels of health facilities and to address factors influencing maternal health behavior and outcomes in an appropriate way. These were among the major factors contributing to the MMR in the Indian states studied [6–8].

‘Social accountability’ has been highlighted as a potential mechanism to improve the performance of the health system in contributing to better maternal health outcomes [10–12]. Social accountability refers to the mechanisms that citizens can use to hold the state and service providers to account for their actions. It aims to improve service delivery through participatory processes to identify health service gaps and women’s needs, and to demand that the health system address these needs [10–12]. It therefore reinforces and legitimizes these demands through intermediaries such as the media or local elected leaders, leading to their better understanding and receptiveness to women’s concerns.

Few studies, however, address social accountability in maternal health in India [10–12]. Overall, they show that social accountability mechanisms lead to better maternal health services, especially for the poor, marginalized and vulnerable groups, through the process of information, dialogue and negotiation. The process helps to empower such groups to demand rights and better services, and also changes the way health providers perceive such groups as genuine rights holders. More evidence is required to analyze if and how social accountability mechanisms improve maternal health. The research reported here seeks to contribute to this evidence through a case study in selected districts of Gujarat state.

Gujarat is situated in western India and is economically one of the highly developed states with a high proportion of the population living in urban areas (> 42%) [13]. Although Gujarat has better maternal health status than the national average (respectively 122 per 100,000 live births compared to 178), it lags behind states like Kerala (66) and Tamil Nadu (90) [4]. The Government of Gujarat has taken several initiatives to improve maternal health services, such as the *Chiranjeevi Yojana* (free childbirth services for poor women in private health facilities)*,* the *Kasturba Poshan Sahay Yojana* (financial assistance for poor pregnant women). There remain, however, challenges with their implementation and uptake [14–16]. Although social accountability interventions aimed at improving maternal health are reported in Gujarat [17, 18], how they operate has not yet been documented.

This study explores the existing social accountability mechanisms for maternal health, the factors they address and how the results of these mechanisms are perceived.

### Conceptual framework

**Fig. 1:**
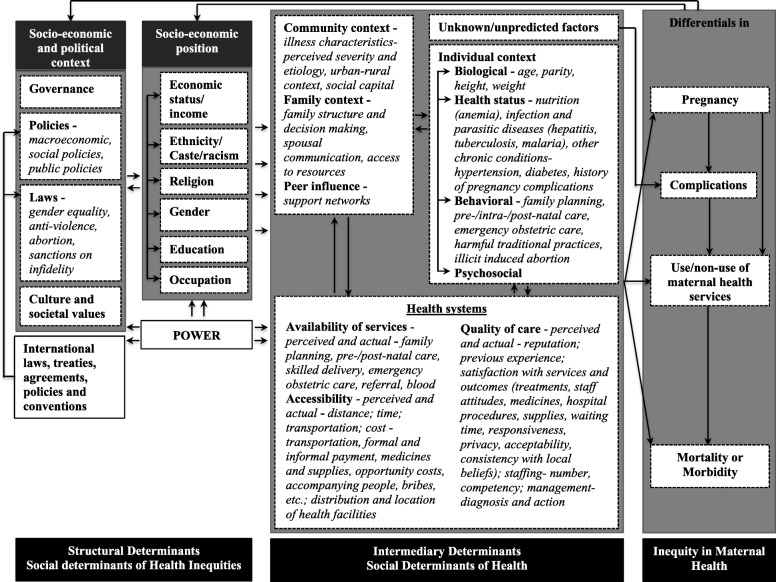
Conceptual framework of social determinants of maternal health

Various contextual factors influence access to and use of maternal health services and contribute to maternal health inequities [19]. Different frameworks have been used to study the factors influencing maternal health such as the 1994 Thaddeus and Maine’s ‘three-delay model’ [20], the 1992 McCarthy and Maine’s framework on distant and immediate determinants of maternal death [21], and the Commission on Social Determinants of Health framework [19, 22]. For this study, we integrated these into one framework to contextualize it for maternal health (Fig. 1).

The framework identifies various factors responsible for maternal health and inequities related to structural and intermediary factors. *Structural factors* are socioeconomic and political contexts that produce socioeconomic stratifications and are particularly responsible for producing inequities (e.g. governance, policies and laws, cultural and social values). A person’s socioeconomic position is determined by economic status, education, ethnicity, religion, etc. *Intermediate factors* include the community and individual context and health system factors contributing to maternal health outcomes. The structural determinants operate through these intermediate factors to produce unequal maternal health outcomes.

In order to understand if social accountability mechanisms can contribute to improved maternal health it is necessary to examine which of these contextual factors these mechanisms influence and in what way.

## Methods

This paper draws on the qualitative research conducted in two adjoining districts in the eastern part of Gujarat – *Dahod* and *Panchmahal.*

### Study area

The districts were chosen based on the working areas of *SAHAJ* – a civil society organization (CSO) that makes interventions in social accountability for maternal health in the districts in partnership with *ANANDI* – a community-based organization (CBO). The social accountability mechanisms analyzed in this study are not limited to those implemented by *SAHAJ*, but include all such mechanisms in the districts identified through the data collection.

The districts lag behind in socioeconomic and maternal health status compared to the state (Table 1), with a higher percentage of poor, rural, illiterate and socially disadvantaged groups, like SC/ST, than the state-level aggregate. These groups are disadvantaged in terms of the uptake of maternal health services in Gujarat [23], and are hereafter referred to as the disadvantaged group. The working areas of the CSOs cover 25 villages under four Primary Health Centers (PHCs) in the two districts. Since the districts share a similar socioeconomic and maternal health status for most of the indicators compared to national average, these were considered as one cluster.

**Table 1:** State versus district indicators on socio-demographic and maternal health status

Socio-demographic and maternal health indicators	Gujarat	Dahod	Panchmahal
Mothers who had antenatal check-up in first trimester (%)^a^	73.9	49.2	60.0
Institutional birth (%)^a^	88.7	84.3	79.6
Mothers who received postnatal check-up within two days of delivery (%)^a^	63.4	57.9	54.0
Pregnant women (15–49 years) who are anemic (< 11.0 g/dl) (%)^a^	51.3	64.6	57.0
Female literacy rate (15–49 years) (%)^a^	72.9	52.2	64.7
Scheduled Castes (%)	6.7^b^	2.0^c^	4.2^c^
Scheduled Tribes (%)	14.8^b^	74.3^c^	30.2^c^
Households below the poverty line (%)	16.6^d^	31.8^c^	31.2^c^
Urban population (%)	42.6^e^	9.0^c^	14.0^c^

^a^ National Family Health Survey - 4; ^b^ calculated from data obtained from: http://censusindia.gov.in/2011census/dchb/Gujarat.html; ^c^ District Census Handbook, 2011; ^d^ Planning Commission, 2014; ^e^ Census of India 2011 (Provisional population totals)

Gujarat has followed India’s three-tier system to provide primary health care at the village level, secondary care at the sub-district and district levels, and tertiary care at the regional level [15, 24, 25].

### Study participants, sampling and data collection

Respondents included: *policy advisors* or those who were involved in drafting national- and/or state-level health policies; *health providers* (*health professionals*, such as Medical Officers and Female Health Workers (FHWs); *Accredited Social Health Activists* (ASHA)), and *health managers* or Block Health Officers (BHOs); *clients/citizens* (CSOs, such as NGOs, CBOs and women’s groups); *locally-elected representatives* or members of the *Panchayat*; and *clients*/*beneficiaries* or pregnant and new mothers (those having given birth within 2 years before the time of data collection). Participants for the study were chosen purposively in consultation with *SAHAJ* to include people at different levels of the health system and beneficiaries in their working areas. The policy advisors were from civil society and were involved in making the national and state-level policies, such as the National Rural Health Mission (NRHM), the *Chiranjeevi Yojana*. Four focus group discussions (FGDs) were conducted (two with members of women’s groups created by *ANANDI* and two with pregnant and new mothers) from the two districts. Details of the in-depth interviews and FGDs are shown in Table 2. The data were collected between December 2014 and February 2015.

**Table 2:** Data collection detail

In-depth interview	Number of participants
Policy advisor	2 Civil society *policy advisors*
Health provider	*Health professionals*:
	1 Medical Officer
	1 Female Health Worker
	1 *Accredited Social Health Activist*
	*Health manager*:
	1 Block Health Officer
Client/citizen	*Locally elected representatives*:
	2 *Panchayat* members
	*Civil society organizations (CSOs)*:
	2 non-government organizations (NGOs)
	2 community-based organizations (CBOs)
Focus group discussion	
Client/citizen	*Civil society organization*:
	15 members of women’s groups
	*Client/beneficiaries*:
	11 pregnant and new mothers

The in-depth interviews and FGDs were conducted by the first author and three research assistants, who were oriented on the scope of the study at the start. Separate interview guides were developed for each group of respondents. As a part of a multi-state study, pilot interviews were conducted in Uttar Pradesh for the overall study. The tools were adjusted for adequacy, appropriateness and clarity following the pre-testing [26, 27] and then translated in Gujarati language. To ensure that the cultural differences between Uttar Pradesh and Gujarat were taken into account we probed responses to understand their actual meaning within their context. The interviews and discussions were audio-recorded and transcribed and translated into English.

### Data analysis

Data analysis was done using a framework approach [28]. This included coding the data, mapping and organizing data under common themes, and interpretation. A coding framework was developed using codes derived from the research questions and the conceptual framework, and MAXQDA 12 software was used for coding and categorizing the data. We applied triangulation to check consistency of findings for responses from different sources for same questions [29].

## Results

I. Social accountability mechanisms for maternal health

The social accountability mechanisms in maternal health in the study sites can be categorized in terms of *structures* and *activities*. In the following section, we present the structures of social accountability with their respective activities.

The most commonly cited formal structures for social accountability were: 1) *government*-created structures in terms of appointed local-level volunteers and health workers (*Accredited Social Health Activist* (ASHA)*, Female Health Worker* (FHW)), locally elected community representatives and committees (*Sarpanch* and *Panchayati Raj Institution* (PRI) members, and *Village Health and Sanitation Committee* (VHSC)), and 2) *civil society* groups consisting of women’s groups established by the CBO.

**Fig. 2:**
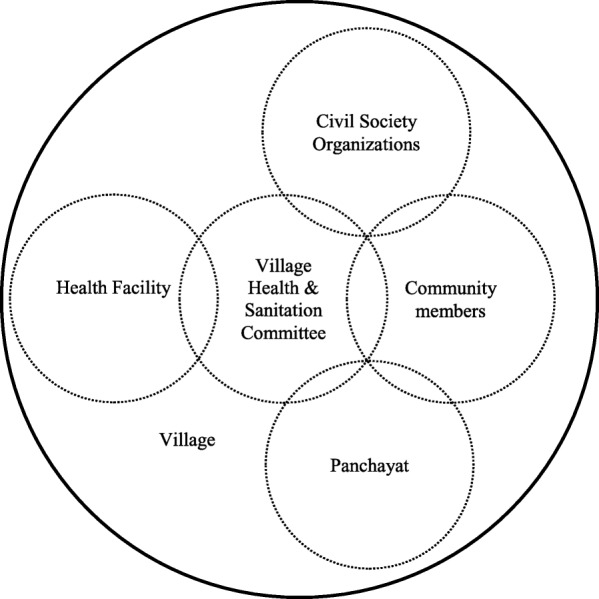
Overview of link between different structures for health sector accountability in a village. Source: Authors Note: the figure shows the links between different actors for health sector accountability at village-level. There are power differences between and within each structure. However, the figure does not show the power differences

Table 3 provides an overview of social accountability mechanism in maternal health in Dahod and Panchmahal. Figure 2 shows the relationship between different structures for health sector accountability in a village.

**Table 3:** Social accountability mechanisms for maternal health in two studied districts of Gujarat

Structure	Description	Expected roles related to social accountability
*Government*		
Accredited Social Health Activist (ASHA)	Community health volunteers selected locally in communities	- Raise awareness among communities about health services, and government health schemes and entitlements through home visits, meetings, etc. - Mobilize communities to claim their health entitlements - Link communities with health system to communicate their concerns
Female Health Worker (FHW)	Auxiliary Nurse Midwives based at Health Sub-Centre – the lowest level health facility	- Provide information about health services, and government health schemes and entitlements to communities during antenatal clinics and their facility visits - Link communities with health system to communicate their concerns
Gram Panchayat or *Panchayat*	Locally elected village council headed by a *Sarpanch*	- Inform communities about health services, and government health schemes and entitlements - Listen to communities’ health needs and concerns in periodic village meetings (*Gram Sabhas*) and assist to resolve them - Link communities with government and health system to communicate their needs and concerns - Hold local-level health facilities accountable towards communities’ needs and concerns
Village Health and Sanitation Committee (VHSC)	Village-level health committee, a sub- or standing committee of *Panchayat*, including members from the health facility, CBOs and all community groups in the village	- Inform communities about health services, and government health schemes and entitlements - Act as an avenue for communities to voice their concerns related to health, address them through periodic health and village meetings, and/or communicate them to *Panchayat* and health system - Ensure communities’ participation in planning, implementation and evaluation of local-level health plans through adequate representation of all community groups
*Civil society*		
*ANANDI* and its women groups – *Dai Sangathan* and *Devgadh Mahila Sangathan*	CBO oriented towards building a community of women, especially disadvantaged groups, to identify their collective needs and concerns, including health, and mobilize them to demand accountability from the government	- Raise awareness in communities about health services, government health schemes and entitlements - Empower women’s groups to identify their collective health issues and demand accountability from government through activities such as awareness raising, capacity building, networking, monitoring and supervision - Mobilize women’s groups to identify their collective needs and concerns related to maternal health through activities such as women’s group meetings, community monitoring and maternal death review, and demand accountability from health system through activities such as protests, dialogues and negotiations with the health system and government

### Government-created structures

1. Accredited Social Health Activist (ASHA) and Female Health Worker (FHW)

Many respondents mentioned ASHAs and FHWs as structures for communities to share and communicate their concerns and issues about health services to the formal health sector. ASHAs are part of the National Rural Health Mission (NRHM) established in 2005 as community health volunteers selected by communities and trained to provide preventive, promotive and basic curative care [30]. In addition, ASHAs are also supposed to create awareness, mobilize and enable communities to claim entitlements from the government. FHWs are Auxiliary-Nurse Midwives based at the Health Sub-Center – the lowest level of health facility in India – responsible for providing health care at the village level, including conducting Village Health and Nutrition Days (VHND) or *Mamta Divas*.

The respondents commonly identified ASHAs and FHWs as sources of information about health services and government schemes and entitlements. They usually provide such information during the VHNDs*,* group discussions at *Anganwadis*^1^ or by visiting the women at their homes. Women’s group members and *beneficiaries* said that ASHAs also assist women to obtain their entitlements by helping them to fill out forms and open bank accounts.

Even though the ASHAs and FHWs were identified as communicating communities’ concerns to the health system, the discussions with the beneficiaries and women’s group members indicated that women approached them mainly about health problems and less often shared concerns about health services.

Beneficiaries mentioned not expressing any concerns to ASHAs or anyone else due to lack of awareness about their rights and entitlements or feeling helpless, as indicated by the following quotes:

‘*How can we say? They can give only after it comes from outside. We cannot ask. We cannot forcefully ask. [..] We take whatever the government gives.’* [Beneficiary].

*‘We are afraid to complain. We do not want to oppose. We do not want to be eyesores.’* [Beneficiary]

2. Sarpanch and Panchayat members

The NRHM policies highlight the empowerment and involvement of the *Panchayati Raj Institutions* (PRI) to ensure the accountability of the public health system through community-led action [31]. *Gram Panchayat* (henceforth *Panchayat*) is a council of locally elected village members as the lowest unit, *Panchayat Samiti* is at block level and *Zilla Parishad* is at district level. The *Panchayat* is headed by a *Sarpanch*, the contact person between the government and the village. *Gram Sabhas* or the periodic village meetings conducted by the *Panchayat* are the main platform for community members to voice their concerns and to participate in planning, implementation and monitoring of development activities, including health [32].

The *Panchayat* members mentioned that *Gram Sabhas* are held regularly in all villages and everyone is invited to participate. Their frequency varied from every 2 to 3 months in Dahod to 3 to 6 months in Panchmahal. A *Panchayat* member from Dahod mentioned that women and men participate in the meetings.

Some of the respondents commented on roles of the *Panchayat* in ensuring the accountability of the public health system. Women’s group members mentioned going to *Gram Sabhas* with women’s issues, including concerns about health services. The *Panchayat* members indicated that people voice complaints in the *Gram Sabhas*, and that the *Panchayat* advises the health authorities at PHC and Health Sub-Centers on issues, such as to refrain from using abusive language in addressing patients. They also mentioned referring complaints about health services, such as corruption and illegal charges by health facilities, to higher authorities.

The *Panchayat* members described creating awareness among women and their families to use health services and secure their government entitlements, and mentioned conducting rallies against corruption and that they are fighting for government entitlements to include people who do not have below poverty line (BPL) cards. A *Panchayat* also mentioned conducting reviews of women giving birth at the government hospital in the *Gram Sabhas*.

In addition, a *Panchayat* member explained joining *ANANDI*’s women’s group, which made her aware of the health services, including maternal health services, schemes and entitlements from the government. The member also mentioned freely discussing complaints and issues in the women’s group.

It remains unclear, however, to what extent beneficiaries really use these representatives, as some beneficiaries from Panchmahal commented that they did not attend any *Gram Sabhas*:

‘*There are Gram Sabhas. But, no man or woman go to there. No one comes to tell us.’*

Moreover, the *Panchayat* members mentioned that they lack training, and that the *Panchayat* faces challenges regarding lack of funding and a lack of transparency of funds spent by its members. Further, *Panchayat* members are powerless and face threats when they demand government accountability, as illustrated by what one respondent said:

*‘Sarpanch is powerless. All Sarpanch are under duress (threat). They cannot voice their grievances against government.’*

3. Village Health and Sanitation Committee (VHSC)

The VHSC is formed at the village level as a sub-committee of the *Gram Panchayat* with members from the *Panchayat*, health facility and proportionate representation of all hamlets and community groups in the village [30, 31]. The NRHM defines the VHSC as a primary institutional mechanism to inform the community about health programs and government initiatives, to voice concerns regarding access to health services, and to participate in planning and management of the health programs.

Respondents mentioned that VHSCs exist everywhere and include members from all sections of the community. The committee usually meets every month in Panchmahal, while in Dahod it was said to meet on a quarterly basis.

A Medical Officer and staff member of a CBO mentioned that there are discussions on all aspects of health in the VHSC meetings on topics such as epidemics and outbreaks in communities, and providing support to health facilities and VHNDs. According to the respondents, the VHSC contributes to social accountability only by making communities aware of maternal and child health issues when mothers and children died. The NGO staff indicated a lack of ownership among the VHSC members, especially the *Panchayat* members, as a major challenge as these are the main formal structure to implement social accountability activities.

Responses from the *Panchayat* members indicated their lack of clarity about the composition and roles of the VHSC. The civil society respondents (policy advisor, NGO staff and CBO staff) expressed the concern that the VHSCs are not active, and that there is a lack of initiatives on the part of the government and CSOs. They also indicated that government efforts to build their capacities jointly with the CSOs were limited to the initial years and only in some villages.

The NRHM highlights the roles of ASHA, *Panchayat* and VHSC in institutionalizing communities’ action to hold the health system accountable, either by communicating their needs and concerns through these structures or directly participating in activities such as *Gram Sabhas*.

However, the responses and discussions show that communities have not been able to use these structures to their full potential because they face various difficulties. For example, the VHSC members lack the ownership and capacity to demand accountability and *Panchayat* members do not feel empowered to hold the government actors accountable.

### Groups established by civil society

#### Women’s groups

*ANANDI* has formed several women’s groups, especially among disadvantaged groups, but two appeared to be particularly involved in social accountability in maternal health – *Dai Sangathan*, or group of traditional birth attendants (TBAs), and *Devgadh Mahila Sangathan*, or group of tribal women. Women’s group members mentioned that TBAs are also member of the *Mahila Sangthan*. We refer to these groups as ‘women’s groups’ hereafter. Respondents mentioned that the groups have existed for about 20 years and were formed because of the need for recognition as well as to identify and discuss their concerns and collectively voice them at relevant forums. The *Mahila Sangathan* also has smaller issue-based groups or committees, such as health, education or livelihoods. Each committee has two women from each village.

The group members meet regularly and at least every month to identify and discuss their concerns, including maternal health. Examples are the lack of VHNDs, land ownership, or social security. The members also described visiting women at their homes to discuss such issues. They described first trying to solve the issues collectively within their own groups, and then take the unresolved issues collectively to *ANANDI, Gram Sabha* or other village meetings.

*‘We try by ourselves. If we can’t fix, then we prefer to go to ANANDI [..]. We talk to the Sarpanch also in our village meetings.’* [CSO member].

The group members also mentioned directly discussing their concerns collectively at ANC clinics with ASHAs and FHWs on issues such as lack of care, or making demands collectively at health facilities or at higher levels on issues such as lack of medicines, equipment and supplies. They also reported carrying out protests and rallies from district to central level, and going to Delhi (central level) to protest, for example on food security or social security. Moreover, their other activities, such as creating awareness among women and families about health services and entitlements, monitoring their use of maternal services, etc., contribute to demanding accountability from the health providers.

*ANANDI* provides the necessary training and support to the women’s groups in conducting these activities through monitoring and supervisory visits by their staff. The CBO staff said that they create awareness in the communities, and make demands and conduct dialogues with the district health system along with the women’s groups to demand its social accountability.

Responses indicate that CSOs have played an important role in identifying communities’ concerns, including about health services and making the health system accountable by referring communities’ collective voices to the health system and conducting dialogues and negotiations through the women’s groups and CBO staff.

### Examples of two major social accountability interventions by CSOs

In addition to the meetings and the activities described above, two major social accountability interventions for maternal health are implemented by the NGO jointly with *ANANDI* and their staff and volunteers – *community monitoring* and *maternal death reviews* (MDR).

*Community Monitoring* involves collecting information on maternal health care from all pregnant and new mothers in the villages – in the eighth month of pregnancy for ANC and 10–15 days after delivery for childbirth and postnatal care. The monitoring is done using the Monitoring Quality of Maternal Health Care tool, developed using the NRHM standards and inputs from experts and communities, and adapted to match the low level of literacy in the communities.

The information collected and compiled into periodic *report cards* are to enable the NGOs, CBOs and women’s groups to demand accountability from the health system. The report cards not only show changes in the maternal health status in the communities, but also act as a source of information and evidence in terms of existing maternal health services, the quality of care provided, and gaps in the maternal health care system. The report cards are used during the *Jan Sambads* or public dialogues that the CSOs organize to engage in dialogue with the health authorities at district level and below.

*‘[..] they have now increasing dialogue. This is not only just we share the report card. But, there is constant dialogue of this Sangathan (group) members and the health system [..].’* [CSO member].

#### Maternal death review (MDR)

Respondents said that the CSOs and the health system are carrying out separate MDRs. The CSOs described their MDR as *social autopsy* or review of maternal deaths for all associated causes including social factors, whereas the health system’s *verbal autopsy* focuses primarily on medical causes. The CSOs said they used the information from the MDR to inform communities and the health system about such issues, and demand the latter’s accountability. The NGO and CBO staff described conducting dialogues on issues arising from the MDR with the health sector at health facility and block levels or during the public dialogues in the presence of the women’s groups’ members.

From such MDR, there are further compilations of maternal deaths at state and national levels by the CSOs and their networks to demand accountability. One such example is the *Dead Women Talking* – a national compilation of maternal deaths [33].

Responses indicated that civil society activities have brought about changes in the determinants of maternal health, which we present in the following section.

II. Influence of social accountability mechanisms on maternal health

Changes in the contexts that influenced maternal health outcomes brought about by the social accountability mechanisms were perceived in various ways by different groups of respondents. Using the social determinants framework, changes can be presented in terms of *structural* and *intermediary determinants* of maternal health.

### Structural determinants

#### Socioeconomic and political context

The responses under this category mainly pertain to governance, policy, health beliefs and women’s status or empowerment. The influence on *governance* largely relates to increased awareness, attention and action by the health system on maternal health issues identified through the social accountability mechanisms, especially at district level and below. From the health manager’s perspective, it largely referred to improved reporting from the CBO and women’s groups leading to awareness of health system issues. Health managers and civil society both mentioned improved reporting of maternal deaths, feedback on health workers’ behavior and information on the maternal health status by the women’s group members and CBO staff.

*‘One good thing is that there is honest reporting. ANANDI Sansthan (organization) also helps us in it – it shows us the prevailing picture [..].’* [Health provider].

The civil society respondents explained that their issues were heard and often acted upon. It led to improved district-level governance in terms of initiation of PHC-level meetings, monitoring of the VHNDs by doctors and district-level managers, prioritizing high-risk pregnancies for PHC-based ANC clinics, and so on.

*‘When I went once, we had a dialogue with the THO (Taluka/Block Health Officer) and Medical Officers. So, all the Sangathan (group) women were along with us. And, it was face-to-face, all the issues were [..], I won’t say sorted out, but at least they were presented and partially, some of the issues were sorted out. [..]’* [CSO member].

At the central and state levels, civil society discussions mainly referred to the government’s realization of the need to improve maternal health facilities following the sharing of the compiled MDR reports as illustrated by:

*‘When we took the national report to the Health Minister and met his Advisor we were told to prepare some concrete proposals which we did. [..] So, there was openness till that level.’*

The influence on *policy* changes was mainly discussed by policy advisors. One policy advisor said that CSOs claimed that the Government of Gujarat promoted a public–private partnership (PPP) through the *Chiranjeevi Yojana*. In the response, the state government changed its policy in order to promote childbirth at public health facilities and strengthening these. Another policy advisor described the role played by CSOs in incorporating community-based monitoring (CBM) in the NRHM, but overall the advisors indicated that social accountability efforts have not been able to influence policies.

Some CSOs and a FHW commented that thanks to the CSO activities, there was a change in traditional mindsets at the community level, and an increased preference for modern health care, including going to a health facility to give birth.

The CSOs also commented that the *status of women*, especially those who are associated with the groups, has improved, partly because of the social accountability efforts. Women said that due to their association with groups and various capacity-building activities in the groups, they are empowered and they can talk about any issue with anyone. Similarly, a *Panchayat* member also commented that many group members are vocal and active, and that health facility staff are aware that these women can complain to anyone so they do not make illegal monetary transactions with them.

#### Intermediary determinants

Responses under this category mainly pertained to the community context (especially social capital), peer influence, community and health system collaboration, health behavior and the health system.

#### Social capital and peer influence

Social capital relates to resources, actual and potential, in terms of interpersonal networks and recognition that people use to realize their interests [34]. The women’s groups are formed with women from same backgrounds, for example *TBAs* and *tribal* women. TBAs and some *Panchayat* members also belong to the women’s group or *Devgadh Mahila Sangathan*. The groups provide women the space to identify, discuss and solve shared problems. The group members, the CBO and even some *Panchayat* members identify themselves as one group, collectively discussing and supporting each other regarding maternal health issues or other issues like food security, livelihoods and gender-based violence.

Through the CBOs and the NGO these women’s groups are also linked to wider like-minded networks such as the *Jan Swasthya Abhiyan* or Public Health Movement and *CommonHealth or* Coalition for Maternal-Neonatal Health and Safe Abortion. These are national-level networks of individuals and organizations advocating for health rights, including maternal health. The CSOs explained that through these networks they have been sharing experiences and also learn from similar groups in other districts and states through exposure visits and sharing experiences. They were in dialogue with the health authorities at state and national level on aggregated issues identified from the civil society activities, for example compiling district-level information on maternal deaths and aggregate data at Gujarat state level.

#### Community and health system collaboration

The health system and related NGOs have a common purpose to provide care. All groups of respondents discussed several instances of collaboration and supporting each other to achieve this goal. Health managers and health professionals mentioned appreciating the presence of women’s groups and their contribution, particularly in making contact with a large number of pregnant and new mothers in their community regarding health care, including pregnant women who had not yet registered.

*‘ANANDI Sanstha is a grass-root level worker since long and dais (TBAs) are also there with whose support this has come about.’* [Health provider].

Different groups of respondents described the support the women’s groups provided: the civil society groups described supporting the health system by convincing families and building their trust to use health services, bringing women to health facilities and providing transport. *Panchayat* members explained that the women’s groups protect and provide moral support to FHWs. Health professionals said that the trained TBAs assist government doctors to attend deliveries at PHCs. Discussion and collaboration with FHWs also supported the latter in addressing systemic or workplace issues, such as lack of equipment, work pressures, etc. A CSO member described one instance:

*‘It happened in Jogwane. [..] they went to Mamta Divas (VHND). We observed that there was no BP (blood pressure not measured). I asked [FHW] BP was not done there. “Because I do not have any instrument, then how can I do?” Then I talked to the MO (Medical Officer) that “there is no BP here, but it is necessary to be taken. If it increases or decreases, then there will be problem with behens (women). If the BP is not taken, then how can it be known?” So, next month the BP instrument came.’*

#### Behavioral factor

The CSOs and FHWs particularly mentioned changes in awareness about maternal health care leading to behavioral change among group members as well as women in the communities. Women’s group members said that they traditionally give birth at home, but due to increased awareness through group activities they now send women to the hospital to deliver their babies. *Panchayat* members and CSO staff explained about women’s awareness about their maternal health and changes in modern health-seeking behavior, such as requesting antenatal check-ups as opposed to having to be told to attend, as illustrated by the following quote:

*‘And, in PHC now it has happened that if there is no ANC then they go to PHC themselves and have their check-ups. And, for them what has happened is – “No, we have to take it (antenatal check-up) for us; we have to take it”.*’ [CSO member].

#### Health system

A large number of responses referred to *health system*, and they could be classified as *availability and accessibility of services,* and *quality of care*.

- Availability and accessibility of services

While a medical officer and a policy advisor explained the NRHM and the Gujarat government’s initiatives such as the *Cheeranjivi Yojana* and the free ambulance service enhanced communities’ access to healthcare facilities, the CSOs claimed that their social accountability actions have improved the availability and accessibility of maternal health services to the women in their community. A key discussion element is the influence of the women’s groups and the CBO staff in lobbying and pushing for such changes.

Examples of how they lobbied for greater availability of services largely focused on activation and regularity of the VHNDs and ANC clinics at PHCs*.* Furthermore, ANCs were strengthened by providing all required services, such as vaccinations, measuring blood pressure, height and weight, etc., in line with the national guidelines. Respondents also mentioned that their actions have achieved the development of new health sub-centers, improvements in functioning of PHC, in the blood storage unit, and improving the availability of ASHAs, nurses and doctors, as illustrated by the following quotes:

*‘We took out rally in 2014 for the improvement of Mamta Divas (VHND). [..] Nurse was not coming to Kharedi faliya (village), therefore we had the rally. [..] Kharedi faliya had not applied for the arrangement of a nurse. Therefore, the nurse was not posted. We applied, and the nurse was posted.’* [CSO member].

*‘If there was no reporting then blood-storage unit would not be available. [..].’* [Health provider].

Examples of improving accessibility included cultural acceptability, for example the increasing acceptance and trust in public health facilities and health care among women and community members. Moreover, a changed attitude among health professionals towards group members and not demanding informal payments were mentioned.

b. Quality of care

The general consensus from respondents across all backgrounds is that *the quality of care* has improved largely due to lobbying and monitoring from the women’s groups, the use of the report card by the CSOs, and involvement and response from the government. The VHNDs especially have been associated with improved quality of care.

In addition, discussions on the quality of care referred mainly to the improved behavior of health professionals and the care they provided. Health managers and CSOs mentioned that health professionals have attended regularly, providing proper care, and have been cordial and responsive to the women who visit them.

*‘Earlier what used to happen was that when patients used to come, the Medical Officer or staff would not pay much attention or we realized that they used to take long to attend the patients. So, when we came to know about it we sensitized them about it. So, now if the patient is sitting there they approach them and ask, “Why are you sitting there? Come along”.’* [Health provider].

There was general consensus among all groups of respondents that coverage and use of ANC services, and institutional delivery have increased. The changes in one aspect of the framework brought about changes in the other, and that had the overall effect on the change in coverage and use of maternal health services as illustrated by this quote:

*‘And, not only this, the VHND was also improving, you could see. So, side by side, this (women’s utilization of services) was happening. [..] then we could see that this has a relation. Because of this (VHND) improvement this (service utilization) has improved. I mean VHND, [..] there were services. Women are getting registered, early registration, women are getting all these services. [..].’* [CSO member].

The responses show that respondents across the different groups perceived that the social accountability mechanisms have brought about changes in both the structural and intermediary factors of the framework, leading to improved maternal health in terms of coverage and uptake of services in the study sites.

However, gaps were still reported regarding maternal health in the study sites. Even if the use of ANC services and hospital deliveries have increased, many women continue to give birth at home, often attended by a TBA, and maternal deaths are still being reported. Respondents described challenges particularly in terms of poverty, superstitions, the low status of women, and lack of services and of health professionals at health facilities. Challenges related to existing social accountability mechanisms are that such efforts are limited to certain areas, not all women in the communities who require support are included in the groups, and not all women in the groups are active. Further, some group members also commented not getting adequate responses from the health system to the concerns they raised.

## Discussion

In Gujarat, formal government structures such as ASHA, FHW, *Panchayat* members and VHSC, and civil society structures comprising women’s groups implement social accountability mechanisms in maternal health. However, the civil society structures seem to have the most influence on determinants of maternal health and maternal health outcomes in the two study districts, in particular their community monitoring, MDR and the women’s group meetings. In addition, CSOs use government activities like *Gram Sabhas* to hold the health system accountable.

The social accountability mechanisms were found to influence both the structural and intermediary factors of maternal health. More specifically, the structural determinants that were influenced were governance, policy, health beliefs and women’s status, while the intermediary factors were community contexts like social capital and peer influence, maternal healthcare behavior, and the availability, accessibility and quality of care provided by the health system. There was a general consensus among the respondents that collaboration between the health system and civil society for maternal health has increased considerably.

Changes in structural factors lead to changes in intermediary determinants and vice versa. For instance, improved governance led to better monitoring and response from health managers that further contributed to strengthened health services, such as regular and functional VHNDs. Changes in the communities in terms of valuing modern health care led to greater maternal health-seeking behavior. In some cases, structural factors also seemed to be influenced by intermediary factors. For example, respondents said that the interaction of women’s groups with other groups such as self-help groups (SHGs), has contributed to women’s empowerment in terms of owning land, new sources of *income* or *occupation*, etc.

The social accountability mechanisms were ultimately found to increase the availability, accessibility and quality of maternal health services provided by the government and their uptake by women. Disadvantaged women in particular seem to have benefited as they are the main target groups of the women’s groups. However, more evidence is needed.

### Mechanisms of influence

Our findings corroborate the findings from similar studies on social accountability from Indian and other settings [11, 35–37]. Studies on community score-cards – a social accountability approach similar to community monitoring – illustrates that using these score-cards for dialogues improves relationships between the community and health providers, and improves community empowerment, provider responsiveness, and ultimately enhances the availability, access, quality and uptake of services [35–37].

George describes accountability as a relationship between two unequal partners – communities and health professionals at different levels of the health system [10]. Addressing problems in the provision of health services through accountability mechanisms requires confronting unequal power relations, and critical elements in such undertakings are information and dialogue and negotiation [10].

*Information* in our study addresses two areas. First, it includes creating awareness among the women and their families about maternal health and government entitlements. Being aware is, according to Papp et al., a precursor to generating critical consciousness – and thus enhances people’s ability to participate in dialogues [12]. Second, evidence on health system issues generated through community monitoring and MDR provides information to foster a change in women’s mindsets and to articulate demands.

*Dialogue and negotiation* help to mitigate social biases or prejudices among health professionals and enhance their receptivity to women’s needs [10, 12]. The groups provided women with a sense of agency and collective identity necessary to confront unequal power. The group meetings provide space for members to discuss and reflect on issues that affect their health, and identify collective issues. The groups conducted series of dialogues and negotiations with the health system through meetings and public dialogues, which gave them the opportunity to share their concerns and demands with representatives of the health system. Papp et al. illustrate that such dialogues and negotiations provide a ‘social receptive space’ for women’s groups in which their needs are recognized by health professionals. As George described, the dialogues and negotiations contributed to change in the mindsets of health providers potentially through reflecting on their assumptions about health system issues as well as how they perceived the disadvantaged groups. Such attitudinal change triggered the health system responses to the groups’ concerns and demands.

George identifies legitimacy of the groups and their demands as a critical issue in confronting unequal power. Papp et al. describe how poor and marginalized groups can use their social capital to legitimize their demands, particularly drawing on the social capital that the groups generated through the CBO, NGO and the broader networks’ support. Dasgupta notes that legitimacy can be claimed through the long existence of CSOs as development actors [11], and the existence of the women’s groups for 20 years in the two districts to legitimize the groups and their demands, in addition using the evidence generated through a systematic process.

Our findings on the role and capacity of government structures for social accountability – ASHA and VHSC – corroborate other studies from India [38–40]. Saprii et al. found that ASHAs were mainly performing their role as ‘link worker’ and ‘service extension worker’, but their work as ‘activist’ was less visible. They attributed this primarily to the ASHAs being unaware about what activist means, and their capacity building focused mainly on the other two roles. This might be the case in our study as well. Studies on VHSCs from other parts of India found that while there was equitable representation of all vulnerable groups in the VHSCs, the members were unaware of their roles and responsibilities [39, 40]. The VHSCs also lacked capacity building and supervision from district and block levels.

A critical finding of our study is that accountability mechanisms that support interaction between service providers and their users enhance a systemic and constructive approach that addresses larger systemic issues. For example, discussions of the women’s groups with FHWs on VHNDs led to identification and management of larger systemic issues, such as to the workload of the FHWs and lack of equipment and supplies. Further, as George [10] mentioned, collaborative approaches that promote interactions between the two groups – for example, health providers and communities in this case – benefit both sides. O’Meally highlighted that for social accountability mechanisms to be effective such mechanisms should strengthen state-society alliance/interaction, the responses in our study also showed that collaborative efforts benefitted both health providers and communities [41].

Furthermore, in our study the report cards and reporting of concerns by women’s groups triggered health managers to initiate monitoring visits of VHNDs, and to organize PHC-level meetings. These findings echo Joshi and Houtzager’s view that social accountability triggers horizontal accountability mechanisms [42].

Although in policies the government structures have similar expected roles with civil society, as outlined in Table 3, they are differently implemented. The civil society have managed to involve multi-pronged approach, for example – empowerment and mobilization of the disadvantaged women’s groups through collective processes to confront the health system, intervention at different levels of the district health system, adoption of multiple strategies and forums such as constructive and collaborative interactions with the health sector actors as well as putting pressure through media, public dialogues, etc. But the role of the government structures such as ASHA, *Panchayat* and VHSC were limited mainly to creating awareness and communicating communities’ concerns to the health system because of their limited capacity to perform the social accountability roles and communities did not often use them due to lack of awareness about their accountability roles. Social accountability interventions that take a multilevel and multipronged approach are likely to be effective and successful [41].

#### Policy implications

The influence of social accountability mechanisms was limited to district level and below. Higher-level responses are also required to address health system weaknesses. Joshi and Houtzager, and O’Meally suggested some ways to generate the higher-level (state or political leaders) response [41, 42]. They suggest that CSOs should devise social accountability in such a way that it systematically shifts the incentives for state or political leaders to act in the public interest to strengthen their capacity to promote and/or respond to social accountability; and to enforce legal mechanisms to ensure the political leaders abide by ‘political settlement’ or ‘social contract’ [41]. Dasgupta shared experience in achieving higher-level (state-level) response to monitor a government maternal health program through formation of and advocacy by a wider partnership/network consisting of UNICEF, international NGOs, national organizations and local NGOs [11].

This study highlights the lack of efforts to build capacities of VHSCs as well as their ownership of the community monitoring process. The NRHM has highlighted the role of VHSCs to promote involvement of communities in monitoring of health facilities, including use of service monitoring tools [30]. Shukla et al. shared the experience of generating ownership among elected representatives of the local health plans and better use of available funds for local community priorities through their capacity-building efforts, and involving them in community-based monitoring and planning processes [43]. Such initiatives to build the capacities of VHSCs, including *Panchayat* members, on their roles and responsibilities, and involving them in a community monitoring process, could enhance ownership of such social accountability processes. Further, the study also indicates a need to build capacity of ASHAs to enhance their *activist* role.

Additionally, the government should more frequently and regularly organize social accountability activities such as *Gram Sabhas* encouraging communities’ participation and engaging communities in dialogue and negotiation with government sector actors as a means to enhance constructive collaboration between them in monitoring health sector performance and addressing the performance barriers. This will create opportunities for joint action plans and resources to address health service gaps and improve collaboration when well facilitated. Moreover, strengthening collaboration between the government sector and the civil society in monitoring health sector performance and addressing the performance barriers can enhance social accountability and leverage impact for better maternal health outcomes.

### Limitations of the study

First, the study districts were selected on the basis of the presence of social accountability interventions by civil society. Further, the selection of the respondents for the study and data collection relied on the support of *SAHAJ*. This may have led to potential bias towards positive responses on effectiveness of the CSO-led social accountability mechanisms. However, we included respondents from civil society beyond the CSO staff and women’s group members such as pregnant and new mothers and actors from the government health system from district level and below, and exercised appropriate caution to minimize bias through source triangulation, in interpreting data and through validation from different authors. The responses were consistent across different categories of respondents for most of the findings, and when differences occurred, these were described and interpreted.

Second, the study builds evidence of influence of social accountability mechanisms based on the changes as perceived by the respondents. This does not yet confirm the actual changes. We recommend further research to assess and explain changes due to social accountability.

The third limitation pertains to the representativeness of respondents, particularly the policy advisor and PRI member as respondent groups. We therefore recommend further studies with the policy advisors from the government health system and PRI members to explore influence of social accountability mechanisms on maternal health.

Fourth, there were limited responses on the influence of social accountability mechanisms on some aspects of the social determinants of maternal health framework, such as laws, policies, culture, socioeconomic status and family context. We suggest further studies to explore the influence of social mechanisms on these aspects of the framework.

Lastly, power differences exist between and within different structures of accountability in health sector (Fig. 2), which are crucial to ensure health sector accountability. Any effort to address issues of accountability would require addressing such power-asymmetries. Our study, however, is limited in this aspect and we recommend for further studies to understand the power differences that exist between and within structures of health sector and their influence on health sector accountability.

## Conclusion

To our knowledge, this study is among the first to describe how social accountability mechanisms contribute to improved maternal health outcomes in terms of access to and use of maternal health services. Based on the case of two districts in Gujarat, the study has described existing social accountability mechanisms from both the government and civil society, and presented the perceived changes brought about by the social accountability mechanisms in some structural and intermediary determinants of maternal health.

The study found that social accountability mechanisms, mainly from civil society, were able to influence governance, policy, health beliefs, women’s status, social capital, maternal healthcare behavior, and the availability, accessibility and quality of the health service delivery system. Such factors overall had a positive influence on increased use of maternal health services.

The social accountability mechanisms particularly generate information/evidence about performance of health system and empower disadvantaged women to make collective demands on the health system. The mechanisms promote interaction between the disadvantaged women groups and the health system through dialogue and negotiation, generating changes in the women’s perception of themselves and the health system, mitigating the latter’s biases/prejudices towards the women’s groups and being receptive towards their needs. Such changes ultimately improve relations between the health system and the women’s groups in terms of trust and collaboration, and generate appropriate responses from the health system in meeting the demands of the women’s groups.

The study identified gaps in the social accountability mechanisms in terms of lack of capacity and ownership of the government structures of these mechanisms, and that the influence of the mechanisms is limited to local/district level.

## Abbreviations

ANC: Antenatal Care
ASHA: Accredited Social Health Activist
BHO: Block Health Officer
BPL: Below Poverty Line
CBM: Community-based Monitoring
CBO: Community-based Organization
CSO: Civil Society Organization
FHW: Female Health Worker
MDR: Maternal Death Review
MMR: Maternal Mortality Ratio
NGO: Non-governmental Organization
NRHM: National Rural Health Mission
PHC: Primary Health Center
PNC: Postnatal Care
PRI: Panchayati Raj Institution
SC/ST: Scheduled Caste/Scheduled Tribe
TBA: Traditional Birth Attendant
VHND: Village Health and Nutrition Day
VHSC: Village Health and Sanitation Committee

## Footnotes

1 Literally means ‘courtyard shelter’, and refers to a village center to provide basic maternal and child health care in Indian health system.
